# *BRAF* V600E-Mutant Acute Myeloid Leukemia: A Case Series and Literature Review of a Rare Entity

**DOI:** 10.3390/genes15111383

**Published:** 2024-10-28

**Authors:** Giby V. George, Andrew G. Evans, Audrey N. Jajosky

**Affiliations:** Department of Pathology and Laboratory Medicine, University of Rochester Medical Center, Rochester, NY 14642, USA

**Keywords:** Acute myeloid leukemia, AML, BRAF V600E

## Abstract

**Background:** Although *BRAF* V600E mutations are common in solid tumors and select hematologic neoplasms, they are reported less frequently in myeloid malignancies. Of the cases of *BRAF* V600E-mutant acute myeloid leukemia (AML) that have been described, most display monocytic morphology and concurrent *KMT2A* rearrangement. Strikingly, all cases have been associated with poor survival. **Case Presentation:** Here, we report two cases of AML, one diagnosed in an elderly male with metastatic lung adenocarcinoma and hepatocellular carcinoma and the other diagnosed in a young boy previously treated for B-cell acute lymphoblastic leukemia. Peripheral blood NGS revealed oncogenic mutations in *BRAF* p.V600E (VAF = 33%), *TET2* p.M508Cfs*25 (VAF = 48%), *TET2* p.C211* (VAF = 49%), *ZRSR2* p.R295* (VAF = 71%), *BRAF* p.N581S (VAF = 6%), and *EZH2* c.118-2A>G, p.? (VAF = 4%) in case 1 and *BRAF* p.V600E (VAF = 1%) and *KRAS* p.G12A (VAF = 28%) in case 2. Cytogenetic workup revealed a complex karyotype in case 1 and an abnormal karyotype with non-clonal aberrations and *KMT2A* (*MLL*) rearrangement in case 2. Morphologically, both patients were found to have AML with monocytic features. The post-mortem examination of case 2 also revealed extensive solid organ infiltration, consistent with a monocytic leukemia. Both patients died within days of diagnosis, demonstrating the lethality of this molecular subgroup of AML. **Conclusions:** Our cases add to the literature, highlighting the poor prognosis of patients diagnosed with *BRAF*-mutant AML. Although it is uncertain whether the complex karyotype and somatic mutations observed in case 1 and *KMT2A* rearrangement and variants identified in case 2 may have either independently or cooperatively conferred a poor prognosis, we contend that additional comprehensive studies are needed to further understand the pathophysiology and prognosis of *BRAF* mutations in AML. We further posit whether patients with *BRAF* V600E-mutant AML may benefit from the combined use of BRAF inhibitors and/or RAS-pathway-targeting regimens, which are currently FDA-approved for the treatment of *BRAF* V600-mutant solid tumors and *BRAF*-mutant histiocytic neoplasms.

## 1. Introduction

The most common oncogenic *BRAF* mutation involves the substitution of glutamic acid for valine at amino acid 600 (V600E) in the protein’s activation loop, leading to constitutive downstream activation of MEK/ERK signaling [1]. *BRAF* V600E mutations are common in solid tumors, including melanoma and papillary thyroid cancer, and hematologic neoplasms, such as Langerhans cell histiocytosis (LCH) and hairy cell leukemia (HCL), but infrequent in myeloid malignancies [2]. Although RAS/MAPK pathway mutations affecting *NRAS*, *KRAS*, and/or *PTPN11* are common in de novo or acute myeloid leukemia (AML)-myelodysplasia-related (MR), *BRAF* variants rarely occur in AML [3]. Of the reported cases, most demonstrate monocytic morphology and/or concurrent *KMT2A* rearrangement [1,4,5]. A small number of *BRAF* V600E-mutant AML cases also occur secondary to cytotoxic therapy (i.e., are therapy-related) [4]. Alarmingly, patients with *BRAF* V600E-mutant AML demonstrate short survival (one month following diagnosis) [4,6]. Specifically, when evaluating the role of *BRAF* mutations in AML progression, it was found that compared to patients with de novo AML or those with secondary AML without *BRAF* mutations, patients with *BRAF* mutations showed an incredibly poor prognosis, with a median survival of 126 days (range: 2–290 days, *p* = 0.0012) [7].

Accordingly, here we report two cases of *BRAF* V600E-mutant AML. The first case was diagnosed in an elderly gentleman with metastatic non-small cell lung cancer and hepatocellular carcinoma, while the second occurred in a young child previously treated for B-cell acute lymphoblastic leukemia (B-ALL). Similar to prior reports, both patients died within days of diagnosis, reinforcing the lethality of this AML subgroup. Importantly, in the literature, one patient with *BRAF* V600E-mutated AML demonstrated a dramatic but transient response to combination therapy with the BRAF inhibitor ‘dabrafenib’ and the MEK inhibitor ‘trametinib’ [1]. We posit whether patients with *BRAF* V600E-mutant AML may benefit from the use of BRAF inhibitors and/or RAS-pathway-targeting regimens that are FDA-approved for other *BRAF* V600-mutant malignancies.

## 2. Methods

### Microscopy, Flow Cytometry, Fluorescence In Situ Hybridization, and Targeted DNA-Based NGS

Standard Wright Giemsa stained peripheral blood smears were prepared. The expression of cell surface markers was evaluated by flow cytometry (Navios, Beckman Coulter) using the ClearLLab 10C 10-color Myeloid (M1, M2) panels (Beckman Coulter). A total of 20 metaphase spreads were analyzed by Giemsa (G)-banding for chromosomal analysis. Fluorescence *in situ* hybridization (FISH) was performed on 200 interphase nuclei for both cases. For case 1, the following AML panel probes (Abbott Molecular/Vysis, Inc., Abbott Park, IL, USA.) were used: LSI EGR1 (5q31) SO/D5S23, D5S721 SG; LSI D7S486 (7q31) SO/CEP 7 (D7Z1) SG; LSI RUNX1T1/RUNX1 Dual Color, Dual Fusion Translocation; LSI MLL Dual Color, Break Apart Rearrangement; LSI PML/RARA Dual Color, Dual Fusion Translocation; LSI CBFB Dual Color, Break Apart Rearrangement; LSI 13 (13q14) SG/LSI TP53 (17p13.1) SO; and LSI RARA Dual Color, Break Apart Rearrangement probes. For case 2, interphase FISH was performed using the following ALL probes (Abbott Molecular/Vysis, Inc., Abbott Park, IL, USA.): LSI BCR/ABL1 ES Dual Color Translocation, LSI MLL Dual Color, Break Apart Rearrangement, LSI ETV6(TEL)/RUNX1(AML1) ES Dual Color Translocation, and LSI IgH Dual Color, Break Apart Rearrangement probes. For the diagnosis of AML in case 2, the NUP98 Break Apart Rearrangement Probe (Empire Genomics, Inc., Depew, NY, USA) was used, in addition to the aforementioned probes for RUNX1T1/RUNX1, KMT2A, PML/RARA, CBFB, and RARA.

Targeted DNA-based NGS was performed on the peripheral blood in both cases using the 34-gene Illumina Trusight Myeloid Panel. In case 2, targeted DNA-based NGS was performed post-mortem on the patient’s liver, spleen, and mesenteric lymph node using ThermoFisher’s 35-gene Oncomine Focus Assay. Finally, to identify any additional cases of *BRAF* V600E-mutant hematologic malignancies at our institution, we queried our in-house clinical molecular database (InfoTrack) from 2018 to 2023.

## 3. Results

### 3.1. Clinical Presentation, Diagnosis, Treatment, and Follow-Up

#### 3.1.1. Case 1

A 75-year-old male smoker presented to his cardiologist with shortness of breath. A CT angiography revealed bilateral lung masses (right lower lobe [RLL]: 2.8 × 1.5 × 1.6 cm and left lower lobe [LLL]: 3.6 × 5.1 × 3.8 cm). An endobronchial ultrasound (EBUS)-guided biopsy and the subsequent pathological examination of the lesions revealed multifocal lung adenocarcinoma with no actionable mutations by targeted DNA-based next-generation sequencing (NGS).

Following stereotactic body radiation therapy (SBRT) to the LLL lesion, he presented with anterior uveitis. CT imaging and biopsy revealed an amelanotic ciliary body mass, consistent with metastatic lung adenocarcinoma, which was later treated with RT. Within the span of a few months, PET-CT showed a 4.9 cm liver mass and two occult pelvic osseous lesions (right hemisacrum and left posterior acetabulum), suspicious for further metastases. Following interval growth, biopsy of the hepatic lesion revealed moderately differentiated hepatocellular carcinoma (HCC). Additional bony lesions (within the left humerus, left scapula, and T7 thoracic vertebra) were identified on subsequent PET scans. Systemic therapy with pembrolizumab, carboplatin, and pemetrexed was planned along with Y90 radioembolization of his HCC.

He subsequently presented to the emergency department (ED) with shortness of breath, nausea/vomiting, and general malaise. Laboratory examinations revealed elevated blood urea nitrogen (BUN) (44 mg/dL) and creatinine (3.88 mg/dL) consistent with acute kidney injury, significant thrombocytopenia (platelets: 20 × 10^3^/uL), and leukocytosis (120.5 × 10^3^/uL) (Table 1).

**Figure 1:**
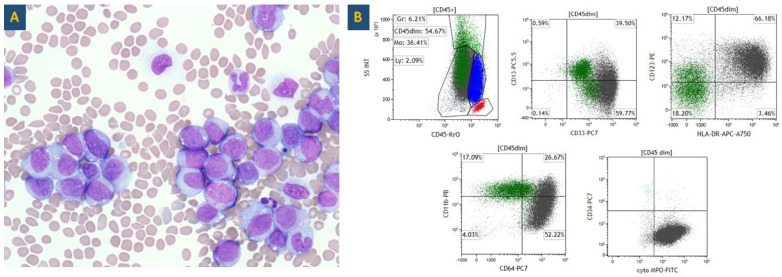
Pathologic examination of case 1. (**A**) Peripheral blood examination (50X oil lens objective) shows abundant medium-to-large blasts with moderate-to-intense basophilic cytoplasm and occasional vacuoles. Nuclei contain finely dispersed chromatin and distinct round-to-oval nucleoli. (**B**) Flow cytometry evaluation of a peripheral blood specimen revealed an expanded CD45-dim gate comprised of an aberrant blast population (displayed in gray, 94% of total analyzed cells) spilling into the granulocytic (green) and monocytic (blue) gates. The lymphocytic gate is depicted in red. The blast population was noted to co-express CD13 (small subset), CD33, CD123, HLA-DR, CD64, CD11b (subset), CD15 (subset), CD38, and cytoplasmic MPO, while being negative for CD34 and all other markers tested.

Peripheral blood examination demonstrated macrocytic anemia with anisocytosis, thrombocytopenia, and abundant circulating blasts (Figure 1A) as well as hypolobated, hypogranular neutrophils. Flow cytometry analysis of the peripheral blood revealed an aberrant blast population, which accounted for 94% of total analyzed cells and co-expressed CD13 (small subset), CD33, CD123, HLA-DR, CD64, CD11b (subset), CD15 (subset), CD38, and cytoplasmic myeloperoxidase (MPO), while being negative for CD34 and all other markers tested (Figure 1B), consistent with a diagnosis of AML.

The patient continued to gradually worsen with new-onset right-sided weakness, concerning for a cerebrovascular accident secondary to leukostasis. Given his rapid deterioration, the patient’s family decided to forego leukapheresis in favor of palliative care. Posthumous chromosome Giemsa (G)-banding demonstrated a complex karyotype, while fluorescence *in situ* hybridization (FISH) revealed a copy number gain of *RUNX1T1* (8q22) in 82% of nuclei (Table 2). Targe ted DNA-based NGS showed oncogenic variants in *BRAF*, *TET2*, *ZRSR2*, and *EZH2* (Table 2).

#### 3.1.2. Case 2

A 2-year-old boy with developmental delay and a secundum atrial septal defect presented to the ED with hypothermia and lethargy. Laboratory examination revealed severe anemia (hemoglobin <3 g/dL), thrombocytopenia (platelets: 19 × 10^3^/uL), and leukocytosis (96.5 × 10^3^/uL). Morphologic and flow cytometry evaluation of the peripheral blood was consistent with a diagnosis of B-ALL. Bone marrow examination showed involvement by B-ALL. Chromosomal analysis revealed hyperdiploidy, with an abnormal hyperdiploid clone identified in three cells harboring extra copies of chromosomes X, 3, 4, 6, 8, 10, 11, 12, 14, 17, 18, 21, and 22, with concurrent loss of chromosome Y (Table 2). Interphase FISH was notable for abnormal copy number gains of the *BCR* locus (22q11.2) in 34% of nuclei, *KMT2A* locus (formerly *MLL*, 11q23) in 40% of nuclei, *ETV6* (12p13) and *RUNX1* (21q22) loci in 37% of nuclei, and *IgH* locus (14q32) in 40.5% of nuclei (Table 2).

The patient’s interval tumor lysis syndrome was treated with rasburicase and allopurinol, after which he was started on induction chemotherapy per clinical trial “AALL1732” with vincristine, daunorubicin, PEG-asparaginase, dexamethasone, intrathecal methotrexate, and cytarabine [8]. A follow-up 28-day bone marrow examination revealed no residual leukemia but markedly left-shifted trilineage hematopoiesis. Subsequent interim maintenance therapy with high-dose methotrexate, vincristine, and 6-mercaptopurine (6MP) was complicated by infections.

A year later, he presented with symptoms concerning for septic shock. Laboratory results were notable for anemia (hemoglobin: 7.5 g/dL), leukocytosis (leukocyte count: 27.2 × 10^3^/μL), and severe thrombocytopenia (platelet count: 16 × 10^3^/μL) (Table 1). CT imaging of the head and neck and chest/abdomen/pelvis revealed endobronchial inflammation and gastrointestinal ileus. He underwent an exploratory laparotomy for abdominal compartment syndrome and subsequently developed multi-organ failure and disseminated intravascular coagulation. Given his worsening condition, his family choose to withdraw care.

**Figure 2:**
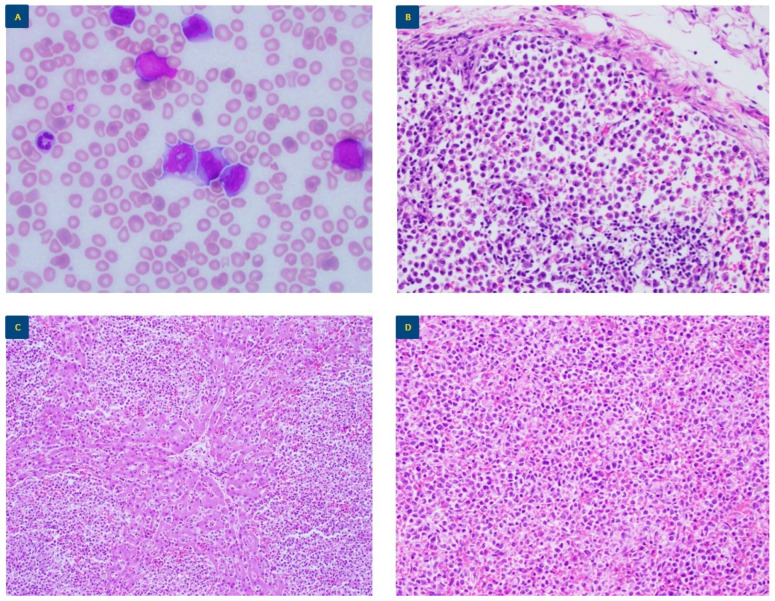
Pathologic examination of case 2. (**A**) Peripheral blood examination (50X oil lens objective) shows scattered immature monocytic forms and occasional blasts. Post-mortem examination revealed diffuse solid organ infiltration by monocytic leukemia involving (**B**) mesenteric lymph node, (**C**) liver, and (**D**) spleen (all images at 20× lens objective).

Posthumous peripheral blood (Figure 2A) and flow cytometry evaluation showed abundant atypical monocytes, raising concern for a myelomonocytic malignancy. Chromosomal analysis revealed two related abnormal cell populations. The first clone (stemline) demonstrated a balanced translocation of t(9;11) in four metaphase spreads. The second clone, involving 14 metaphase spreads, harbored a complex karyotype with the following clonal aberrations [in addition to t(9;11)]: a copy number gain of chromosome 22 and a derivative of chromosome 6 with a fragment distal to 11q13 translocated to 6p23. The remaining two cells did not demonstrate any clonal aberrations (Table 2). Interphase FISH was notable for *KMT2A* (11q23) rearrangement in 92% of nuclei. Targeted DNA-based NGS revealed oncogenic variants in *BRAF* and *KRAS*. Post-mortem examination later revealed diffuse solid organ infiltration by monocytic leukemia involving the mesenteric lymph nodes, liver, and spleen (Figure 2B–D). In light of the extensive organ involvement by monocytic leukemia, representative sections of his liver, spleen, and mesenteric lymph node were sequenced. Targeted DNA-based NGS revealed the presence of the *BRAF* V600E mutation in the spleen and lymph node samples at identical VAFs (1%). Additionally, a *KRAS* variant (at a VAF comparable to that detected in the peripheral blood) and an additional mutation in *RAF1* were detected in all three autopsy specimens.

To identify any additional cases of *BRAF* V600E-mutant AML, we queried our in-house molecular database from 2018 to 2023 for *BRAF* V600E-mutant hematologic malignancies. Of 1600 hematologic malignancies sequenced over this time period, 1% (16/1600) were found to be *BRAF*-mutant, including the two AML cases (12.5%, 2/16) described here, as well as patients with HCL (62.5%, 10/16), chronic lymphocytic leukemia (12.5%, 2/16), a malignant histiocytic neoplasm (6.25%, 1/16), and multiple myeloma (6.25%, 1/16).

## 4. Discussion

*BRAF* V600E-mutant AML is exceedingly rare and all cases published to date report poor survival. In this series, we describe two additional cases of *BRAF* V600E-mutant AML. The rapid demise of both patients highlights the dismal prognosis of *BRAF* V600E-mutant AML.

Given the molecular profile and morphologic dysplasia observed in case 1, the patient’s AML likely developed secondary to therapy (for his prior two solid tumors) or may have been myelodysplasia-related (MR), i.e., developing from a preceding myelodysplastic neoplasm or myelodyplastic/myeloproliferative neoplasm (MDS/MPN) such as chronic myelomonocytic leukemia (CMML). Accordingly, the genomic landscape of MDS and MDS/MPNs includes mutations in genes affecting epigenetic regulation (*TET2*, *DNMT3A*), histone modification (*ASXL1*, *EZH2*), RNA splicing (*SRSF2*, *SF3B1*, *U2AF1*, *ZRSR2*), signal transduction (*NRAS*, *KRAS*, *CBL*, *PTPN11*, *JAK2*), and nucleosome assembly (*SETBP1*, *RUNX1*) [3]. In retrospect, the patient is noted to have had persistent macrocytic anemia (MCV ranging from 94 to 100 fl), thrombocytopenia (platelet count ranging from 73 to 101 × 10^3^/μL), and absolute monocytosis (ranging from 0.9 to 7 K/μL) over the span of five years, suggestive of a prior underlying myeloid malignancy. Additionally, the VAF of his *BRAF* V600E variant (33%) is lower than the VAFs of his two *TET2* mutations (p.M508Cfs*25 at 48% and p.C1211* at 49%), supporting the notion of clonal evolution.

The patient described in case 2 developed AML within a year following treatment for B-ALL. Although hyperdiploidy in B-ALL is known to confer a favorable prognosis [9,10], the patient continued to progress despite initial treatment response. His AML was characterized by multiple cytogenetic aberrations, including *KMT2A* rearrangement (Table 2), as well as oncogenic mutations in *KRAS* and *BRAF*. In an attempt to understand whether the *BRAF* V600E-mutant subclone had a propensity to home to any particular organ, which may have contributed to the patient’s organ failure, representative sections of his liver, spleen, and mesenteric lymph node were sequenced. While the *BRAF* V600E mutation was not observed in his liver specimen (although likely present at a <1% VAF) it was identified in the spleen and lymph node at nearly comparable VAFs (Table 2). *KRAS* and *RAF1* variants were also identified in all three specimens.

Although the *BRAF* V600E mutation in case 2 is sub-clonal, the monocytic morphology, as was observed in case 1, and concurrent *KMT2A* rearrangement are consistent with published findings. Previously, Christiansen et al. described three cases of *BRAF* V600E-mutant AML, all of which occurred secondary to cytotoxic therapy, exhibited monocytic morphology, and harbored t(9;11)(p22;q23) *KMT2A* rearrangements [4]. Additional cases of *BRAF* V600E-mutant AML have been described [1,5], all with monocytic differentiation. However, while the case of *BRAF* V600E-mutant therapy-related AML described by Wander et al. possessed a *KMT2A* rearrangement, the four cases reported by Xu et al. arose de novo and lacked this cytogenetic finding [1,5]. Salek et al. describe a pediatric patient initially diagnosed with high-risk LCH who progressed to *BRAF* V600E-mutant AML despite treatment [11]. Although the histiocytic and AML samples shared the *BRAF* V600E mutation with comparable VAFs, the AML possessed additional aberrations including somatic variants in *KRAS*, *NRAS*, *EZH2*, and *IKZF1*, *RUNX1*::*POU2F2* fusion, and monosomy 7 [11]. In a recent single-institution experience, Fei et al. corroborate these findings, reporting V600E to be the most common variant (28.6% [4/14]) among *BRAF*-mutant myeloid neoplasms [7]. In addition to concurrent monocytic morphology and *KMT2A* rearrangement, they report frequent co-mutations in *TET2*, *ASXL1*, and *JAK2* [7].

Recent reports suggest BRAF-mutated AML to be particularly enriched in the setting of AML-MR [12]. Accordingly, in a recent retrospective analysis of *BRAF*-mutant myeloid neoplasms (AML, MDS, CMML, MPNs) over the span of ten years, Abuasab et al. identified a total of 48 patients, 18 of whom were diagnosed with *BRAF*-mutant AML (secondary AML in 66.7% [12/18]) of cases) [13]. Interestingly, they found G469A to be the most frequent *BRAF* variant (25% of patients), followed by V600E in 19% of patients [13]. Unlike V600E mutations, which affect the protein activation loop, G469A mutations impact the P-loop and alter ATP binding, likely conferring BRAF inhibitor resistance [13]. Other cases of non-V600E *BRAF*-mutant AML have also been described [12,14,15]. Regardless, *BRAF* mutations in AML are reported to be independently associated with a poor prognosis, irrespective of clonal burden [4,6,12].

Thus, *BRAF* V600E-mutant AML likely represents an especially lethal molecular subgroup. Although in this study, it is uncertain whether the complex karyotype and somatic mutations in case 1 and *KMT2A* rearrangement and multiple variants in case 2 may have either independently or cooperatively conferred a poor prognosis, we contend that additional comprehensive studies are needed to further understand the pathophysiology and prognosis of *BRAF* mutations in AML. Importantly, elucidating the cytogenetic and molecular mechanisms that give rise to the evolution of *BRAF*-mutant myeloid neoplasms may enable the development of novel treatment approaches for patients [13]. Although monocytic morphology lacks specificity [16], we posit whether patients with *BRAF* V600E-mutant AML may benefit from the combined use of FDA-approved BRAF inhibitors and/or RAS-pathway-targeting regimens.

## Figures and Tables

**Table 1:** Laboratory Investigations.

Relevant Laboratory Investigations at AML Diagnosis	
Case 1	Case 2
BUN: 44 mg/dL Creatinine: 3.88 mg/dL Platelet count: 20 × 10^3^/uL Leukocyte count: 120.5 × 10^3^/uL	Hemoglobin: 7.5 g/dL Platelet count: 16 × 10^3^/uL Leukocyte count: 27.2 × 10^3^/uL

**Table 2:** Cytogenetic and Molecular Workup.

s	Diagnosis	Karyotype	FISH	DNA-based NGS
1	AML	48–49, XY, +8, +18, +2–3mar, inc[10]/46, XY[10]	Copy number gain of *RUNX1T1* (8q22) (82% of nuclei)	*BRAF* c.1799T>A, p.V600E (Variant allele frequency [VAF]: 33%) *TET2* c.1522delA, p.M508Cfs*25 (VAF: 48%) *TET2* c.3633t>A, p.C1211* (VAF: 49%) *ZRSR2* c.883C>T, p.R295* (VAF: 71%) *BRAF* c.1742A>G, p.N581S at 6% (VAF: 6%) *EZH2* c.118-2A>G, p.? (VAF: 4%)
2	B-ALL	58<2n>, X, +X, −Y, +3, +4, +6, +8, +10, +11, +12, +14, +17, +18, +21, +21, +22[3]/46, XY[17]	Abnormal copy number gains of *BCR* locus (22q11.2) in 34% of nuclei, *KMT2A* locus (formerly *MLL*, 11q23) in 40% of nuclei, *ETV6* (12p13), *RUNX1* (21q22) loci in 37% of nuclei, and *IgH* locus (14q32) in 40.5% of nuclei	Not performed
	AML	46, XY, t(9;11)(p21;q23) [4]/ 46–47, idem, der(6)t(6;11) (p23;q13), +22 [cp14]/ 46, XY[2] Non-clonal: add(7)(p14) del(16)(q21)	Abnormal with one to two *MLL* fusion signals, one isolated 5′ *MLL* signal and one isolated 3′ *MLL* signal in 92% (184/200) of interphase nuclei, suggestive of *MLL* (11q23) rearrangement	Peripheral blood: *BRAF* c.1799T>A, p.V600E (VAF: 1%) *KRAS* c.35G>C, p.G12A (VAF: 28%) Solid organs: Liver: *KRAS* c.35G>C, p.G12A (VAF: 23%) *RAF1* c.770C>T, p.S257L (VAF: 29%) Spleen: *BRAF* c.1799T>A, p.V600E (VAF: 1%) *KRAS* c.35G>C, p.G12A (VAF: 21%) *RAF1* c.770C>T, p.S257L (VAF: 23%) Mesenteric lymph node: *BRAF* c.1799T>A, p.V600E (VAF: 3%) *KRAS* c.35G>C, p.G12A (VAF: 18%) *RAF1* c.770C>T, p.S257L (VAF: 21%)

## Data Availability

No new data were created or analyzed in this study. Data sharing is not applicable to this article.
